# High-throughput microfluidics and machine learning-assisted screening of lipid nanoparticle formulations for siRNA delivery

**DOI:** 10.1016/j.mtbio.2026.103483

**Published:** 2026-07-22

**Authors:** Mingzhi Yu, Luhan Wang, Dongsheng Liu, Jianqiang Guo, Jiahao Liu, Jiawei Zhou, Allen Mathew, Zhonglei He, Jing Wu, Nan Zhang

**Affiliations:** a Centre of Micro/Nano Manufacturing Technology (MNMT-Dublin), School of Mechanical & Materials Engineering, University College Dublin, Dublin 4, D04 V1W8, Ireland; b Department of Aerospace and Mechanical Engineering, South East Technological University, Carlow, R93 N9R3, Ireland; c The Centre for Research and Enterprise in Engineering (engCORE), South East Technological University, Carlow, R93 N9R3, Ireland; d School of Medicine, Anhui University of Science and Technology, Huainan, 232001, China; e Key Laboratory of Industrial Dust Deep Reduction and Occupational Health and Safety of Anhui Higher Education Institutes, AUST, Huainan, 232001, China; f Institute of Precision Medicine, School of Medicine, Anhui University of Science and Technology, Huainan, 232001, China

**Keywords:** Lipid nanoparticle formulations, High throughput microfluidics, Machine learning, siRNA delivery, Formulation screening

## Abstract

Lipid nanoparticles (LNPs) are among the most advanced non-viral carriers for RNA delivery; however, the optimization of LNP formulations remains challenging due to the vast and multi-dimensional formulation space. Here, we established a high-throughput microfluidic and machine learning-assisted workflow for stepwise LNP formulation prioritization for siRNA delivery. A library of 864 candidate formulations was generated, and 203 randomly selected empty LNPs were experimentally characterized. Machine learning models were then used to predict particle size and PDI across the full formulation library, supporting the selection of 25 candidates for siRNA loading and biological validation. The results showed that the size have positive relationship with lipids concentration, and the PEG ratio have negative relationship with size, and positive with PDI. After siRNA loading, all selected formulations-maintained particle sizes below 150 nm. In vitro gene silencing results showed that SM-102- and CKK-E12-based formulations achieved stronger S100P knockdown, with relative S100P expression levels reduced to 0.0744 and 0.0611, respectively. In vivo biodistribution analysis further showed that CKK-E12-based LNPs exhibited higher lung signals compared with the other three ionizable lipid groups. These results indicate that favorable physicochemical properties are necessary but not sufficient to ensure optimal biological performance, highlighting the importance of biological validation after model-assisted screening. Overall, this workflow provides a practical strategy for integrating high-throughput formulation preparation, model-assisted prioritization, and biological validation for siRNA-LNP development.

## Graphical abstract

**Figure:**
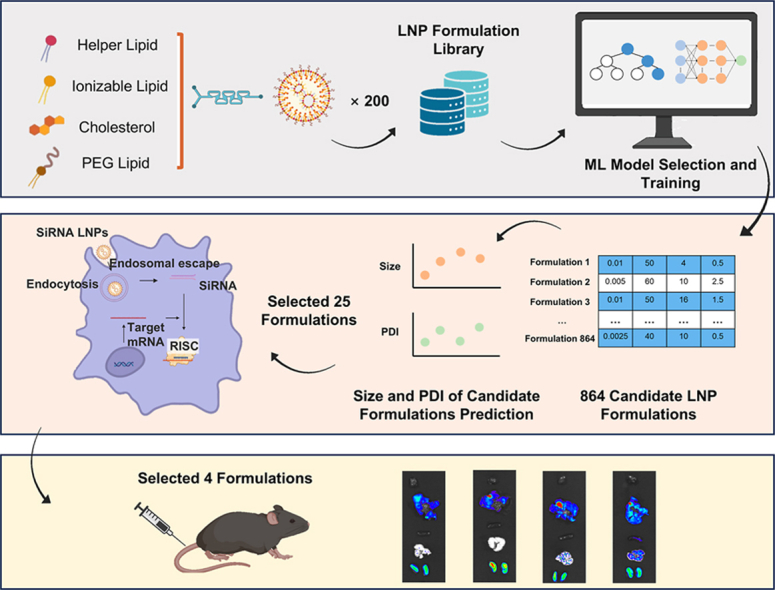

## 1 Introduction

Lung cancer remains one of the most common and deadly cancers worldwide, accounting for an estimated 2.5 million new cases and 1.8 million deaths in2022 [[1], [2], [3]]. For patients with early-stage lung cancer, surgical resection, adjuvant therapy, targeted therapy, and immunotherapy are commonly recommended treatment strategies [1]. However, many patients are diagnosed at an advanced or metastatic stage because early symptoms are often mild, non-specific, or absent [4]. For metastatic lung cancer, systemic treatments such as chemotherapy, targeted therapy, and immunotherapy play a central role, but chemotherapy and immunotherapy clinical efficacy can be limited by systemic toxicity, drug resistance, and insufficient tumor-specific accumulation [1,5]. Therefore, targeted delivery systems have attracted increasing interest as strategies to improve therapeutic efficacy while reducing off-target side effects.

Various non-viral delivery platforms, including protein-based nanoparticles, polymeric nanoparticles, inorganic nanomaterials, PEGylated systems, and lipid-based carriers, have been investigated for lung targeted delivery [[6], [7], [8]]. Among these, lipid nanoparticles (LNPs) have emerged as one of the most clinically advanced nanocarrier systems, particularly following their successful application in COVID-19 mRNA vaccines [9,10]. LNPs are typically composed of an ionizable lipid, helper lipid, cholesterol, and PEG-lipid [11,12]. The ionizable lipid plays a key role in nucleic acid encapsulation and endosomal escape, while helper lipids, cholesterol, and PEG-lipids contribute to particle stability, membrane structure, circulation behavior, and biodistribution [13]. Although considerable efforts have focused on developing new ionizable lipids to improve delivery performance [14,15], the molar ratio among different lipid components is also a critical factor. Changes in formulation composition can substantially influence physicochemical properties such as particle size, polydispersity index (PDI), and zeta potential, which may further affect cellular uptake, gene silencing efficiency, and in vivo organ distribution. In particular, nanoparticles below approximately 150 nm are generally considered favorable for cellular uptake and tumor-associated delivery [16,17].

Small interfering RNA (siRNA) represents a promising therapeutic modality because it can selectively silence disease-related genes at the post-transcriptional level [18]. The clinical potential of LNP-mediated siRNA delivery has been demonstrated by the approval of Onpattro® (patisiran), the first FDA-approved LNP-based siRNA therapeutic [19]. In the context of lung cancer, S100P is of particular interest because it has been associated with cancer cell migration, invasion, and tumor progression [20,21]. Therefore, siRNA-mediated knockdown of S100P may provide a potential therapeutic strategy for suppressing lung cancer progression. However, efficient siRNA delivery remains challenging because naked siRNA is susceptible to nuclease degradation, has poor cellular uptake, and requires an effective carrier to reach the cytoplasm [22,23]. LNPs offer a promising platform for siRNA delivery, but their performance is highly dependent on formulation composition and manufacturing conditions.

Microfluidic technologies provide a powerful approach for improving the reproducibility and controllability of LNP synthesis [24,25]. Compared with conventional bulk mixing methods, microfluidic mixing enables rapid and controlled solvent exchange, leading to more uniform nanoparticle formation [26,27]. Nevertheless, traditional formulation optimization remains largely dependent on trial-and-error experimentation, which is inefficient for exploring the high-dimensional design space generated by different lipid types and molar ratios [28,29]. Commercial microfluidic systems are commonly based on serial or single-channel workflows and often require relatively large formulation volumes, making them less suitable for high-throughput screening under material-constrained conditions [[30], [31], [32]]. To address these limitations, our previous work developed a high-throughput microfluidic platform with an eight-channel parallelized cartridge, allowing simultaneous preparation of multiple LNP formulations with low sample consumption [33]. This platform provides an efficient experimental basis for generating formulation datasets, but experimentally testing every possible formulation remains time-consuming and costly.

Machine learning (ML) offers a complementary framework for identifying formulation-dependent physicochemical trends and guiding down-selection within large combinatorial design spaces [34,35]. Instead of experimentally preparing all possible formulations, a representative subset can be synthesized and characterized, and the resulting data can be used to predict the properties of the remaining formulation space. This approach is particularly valuable for LNP development because formulation–empty LNPs physicochemical property relationships are often non-linear and difficult to interpret using conventional empirical rules alone. By combining high-throughput microfluidic synthesis with ML-based prediction, it is possible to reduce experimental workload while improving the efficiency of candidate selection.

In this study, we established a high-throughput microfluidic and machine learning-assisted workflow to accelerate the screening and optimization of LNP formulations for siRNA delivery in lung cancer. The workflow integrates parallelized microfluidic synthesis, physicochemical characterization, predictive modelling, siRNA loading, in vitro biological evaluation, and in vivo biodistribution analysis. By using empty LNP characterization as an upstream physicochemical screening step, candidate formulations with smallest size and lower PDI were prioritized before siRNA loading and functional validation. This study provides an integrated strategy combining high-throughput formulation generation, machine learning-guided down-selection, and biological validation for efficient LNP formulation development.

## 2 Materials and method

### 2.1 Materials

SM-102, ALC-0315, DLin-MC3-DMA (MC3), DSPC, DOPC and DMG-PEG2000 were sourced from Avanti Polar Lipids Inc. (Alabama, United States). CKK-E12 was purchased from MedChemExpress (MCE, USA). Cholesterol, ethanol, and sodium acetate buffer were purchased from Sigma-Aldrich (Darmstadt, Germany). A Cy3-labeled siRNA with a non-coding, non-targeting sequence was acquired from GenScript Biotech. DMEM 6249 was purchased from Merck Life Science. Fetal bovine serum (FBS), NucBlue™ Live ReadyProbes™ Reagent, penicillin/streptomycin (p/s), SuperScript™ First-Strand Synthesis System for RT-qPCR, S100P siRNA (Gene ID: 6286, Silencer Select, Cat# AM16708, siRNA ID: 17215), and S100 calcium binding protein P probe (Assay ID: Hs00195584_m1) were ordered from Thermo Fisher Scientific (Waltham, MA). RNeasy Mini Kit was ordered from Qiagen (UK). A549 Cell Line human (RRID: CVCL_0023) was purchased from Merck. RNeasy Mini Kit Size, PDI and zeta-potential were measured by a Dynamic Light Scattering Instrument (DLS, Litesizer DLS 500, Anton Paar, The Republic of Austria). Female C57BL/6J mice (7–9 weeks old) were purchased from SKBS (China) and housed in the Laboratory Animal Center of the School of Medicine, Anhui University of Science and Technology. Animals were maintained under controlled environmental conditions (temperature: 20 ± 2 °C, humidity: 55 ± 5%, 12 h light/dark cycle) with free access to food and water. Animals were randomly assigned to different treatment groups to minimize potential bias. All animal procedures were approved by the Ethics Committee of Anhui University of Science and Technology (Ethics Review Approval No. GZ2024-069) and conducted in accordance with the ARRIVE guidelines. Isoflurane anesthesia was used during all injection and imaging procedures to minimize discomfort.

### 2.2 Design of LNP formulations and experimental selection

Four ionizable lipids (SM-102, ALC-0315, MC3, and CKK-E12) were selected, along with helper lipids (DSPC or DOPC), cholesterol, and DMG-PEG2000 to test how formulations affected LNPs properties. The formulation design was guided by clinically validated molar ratios, such as those used in Onpattro and Moderna LNPs [9,19]. To enable comprehensive exploration of formulation space for AI model training, multiple combinations of lipid concentrations and molar ratios were tested, covering ionizable lipid concentrations from 1.25 to 10 mM. The specific design parameters are summarized in Table 1.

**Table 1:** Lipid composition parameters for LNP formulation screening.

Parameter	Unit	Levels
Ionizable lipid type (ILP)		SM-102, ALC-0315, MC3, CKK-E12
Helper lipid type (HLP)		DSPC, DOPC
Ionizable lipid concentration (ILC)	mM	1.25, 2.5, 5, 10
Ionizable lipid ratio (ILR)	%	40, 50, 60
Helper lipid ratio (HLR)	%	4, 10, 16
PEG-lipid ratio (PEGLR)	%	0.5, 1.5, 2.5

In total, 864 possible LNP formulations were generated based on the combinations of lipid types, molar ratios, and concentrations. To reduce experimental workload and reagent consumption, 203 representative formulations were selected using Latin Hypercube Sampling. This strategy ensured uniform coverage of the formulation space, and the selected formulations were used for experimental screening. The Latin Hypercube Sampling ensured that the 203 selected formulations provided broad and uniform coverage of the formulation space, making them well-suited for training machine learning models to predict particle characteristics across the entire set of 864 formulations. The full list is provided in Table S1 in the supplementary documents.

**Fig. 1:**
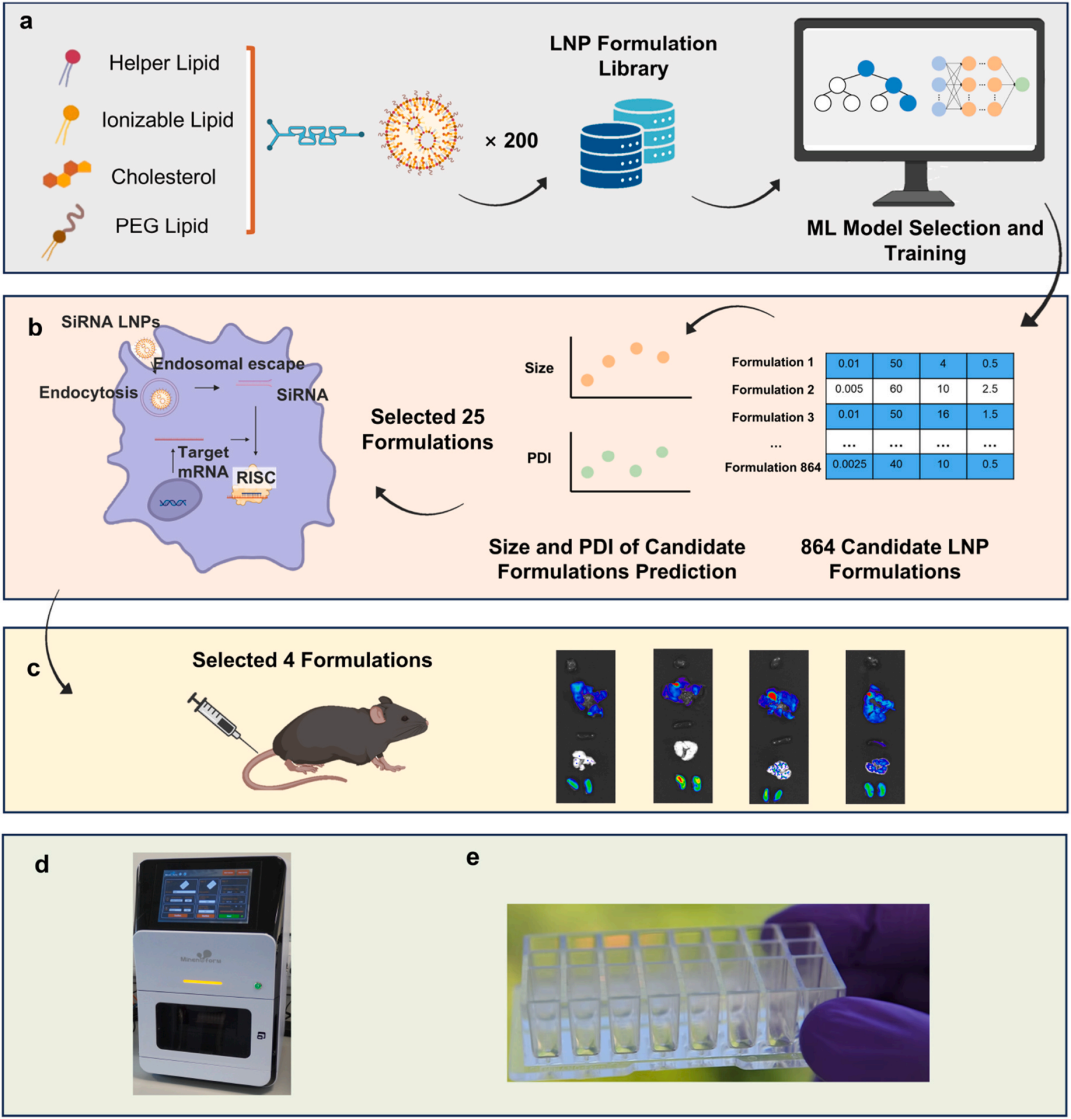
Schematic illustration of high-throughput screening and ML–guided optimization of LNP formulations. (a) Design and synthesis of 203 representative LNP formulations from a library of 864 candidates; (b) ML–based prediction of size and PDI using experimental results from 203 formulations, followed by in vitro screening of 25 selected formulations in A549 cells for siRNA uptake and S100P gene silencing; (c) In vivo biodistribution analysis of four top-performing formulations using Cy3-labeled siRNA in C57BL/6J mice; (d) High-throughput microfluidic device: MiNANO-form; (e) Disposable 8-channel cartridge used with the MiNANO-form.

LNPs were synthesized using a high-throughput microfluidic system (MiNANO-form) and a cartridge (Fig. 1a–d, e). Lipids were dissolved in ethanol and combined according to the experimental design to prepare 130 μL of the organic phase. The aqueous phase consisted of 25 mM sodium acetate buffer (pH 5.0). Prior to each run, the microfluidic cartridges were flushed with ethanol. Then, 300 μL of aqueous phase and 100 μL of organic phase were pipetted into inlets 1 and 2, respectively. The cartridge was loaded onto the MiNano-form, and the two phases were mixed at a total flow rate of 2 mL/min with an aqueous-to-organic ratio of 3:1. The resulting LNPs were collected from the outlet and immediately diluted (1:4) in PBS prior to characterization by DLS (Litesizer DLS 500, Anton Paar, The Republic of Austria) for the size, PDI, and zeta potential.

### 2.3 Machine learning–based prediction and screening of LNP formulation properties

#### 2.3.1 Feature definition and justification

Six formulation-related features were selected for model development, including four numerical variables, ionizable lipid concentration (ILC), ionizable lipid ratio (ILR), helper lipid ratio (HLR), and PEG-lipid ratio (PEGLR), and two categorical variables, ionizable lipid type (ILT) and helper lipid type (HLT). These features were chosen based on their mechanistic relevance to LNP physicochemical properties and their ability to capture the full compositional space. Cholesterol content was treated as a dependent variable, calculated to complete the total lipid to 100%. Feature–output relationships were preliminarily assessed using Spearman correlation analysis with particle size, PDI, and zeta potential.

#### 2.3.2 Model selection, training, and evaluation strategy

To identify the most suitable model for formulation property prediction, four distinct regression algorithms were evaluated: Gradient Boosting Regressor (GBR), Multi-Layer Perceptron (MLP), Support Vector Regressor (SVR), and a Linear Regression (LR) as a baseline. To ensure the highest level of methodological rigor and model generalizability, the dataset was partitioned into a training set (80%) and a hold-out test set (20%). On the training set, a Grid Search with 10-fold cross-validation (CV) was implemented to exhaustively optimize hyperparameters, ensuring that the selected configurations were robust and not overfitted to specific data subsets. The final models, trained using the optimized parameters on the full 80% training data, were subsequently evaluated against the 20% hold-out test set. To eliminate the potential bias introduced by any single random data partition, this entire workflow—comprising random splitting, grid search, and independent testing—was executed across 10 independent iterations with different random seeds. The final performance metrics were reported as the mean and standard deviation across these 10 iterations, providing a statistically robust and unbiased assessment of the models' predictive power on unseen data. Model performance was comprehensively quantified using R^2^, Mean Absolute Error (MAE), Percent Error, and the Spearman Rank Correlation Coefficient. Specifically, the selection criteria were tailored to the physical significance of the target parameters. For LNP size: Emphasis was placed on R^2^, as precise numerical accuracy is essential for ensuring formulation consistency. For LNP PDI: Given that PDI is intrinsically more sensitive to experimental noise, we prioritized the Spearman correlation coefficient. This focus on monotonic relationships and relative ranking—rather than absolute numerical values—reflects the practical goal of identifying formulations with superior monodispersity trends within the design space.

#### 2.3.3 ML workflow for formulation screening and candidate selection

A virtual library of 864 candidate LNP formulations was generated by combining the available lipid types, molar ratios, and concentrations. After identifying the best-performing models, each model was retrained on the full experimental dataset and used to predict the particle size and PDI of every candidate formulation. The predicted values were then combined through a weighted selection criterion that accounts for the relative predictive reliability and practical importance of the two outputs. At this upstream physicochemical screening stage, formulations with smaller actual/predicted particle size and lower actual/predicted PDI were prioritized. This strategy was adopted because siRNA loading can increase particle size; therefore, selecting smaller empty LNPs provides a size margin to reduce the risk that the final siRNA-loaded LNPs exceed the size range generally considered favorable for tumor-associated delivery. In particular, nanoparticles below 150 nm have been reported to facilitate penetration through tumor tissue in animal models [16]. PDI was included as an additional criterion because lower PDI values indicate a more uniform particle population. seven ALC-0315-based formulations and the top six formulations from each of the other three ionizable lipid groups were selected for subsequent in vitro performance evaluation.

### 2.4 Cellular uptake evaluation using Cy3-labeled siRNA

To evaluate cellular uptake, representative formulations were selected from the LNP library based on model predictions (Fig. 1b). For each ionizable lipid, the 25 formulations were chosen, and LNPs encapsulating Cy3-labeled siRNA were synthesized using the MiNANO-form platform. Cy3-siRNA was diluted in 25 mM sodium acetate buffer to final concentrations of 2 nmol/mL (high) and 0.67 nmol/mL (low), and used as the aqueous phase. Cy3-siRNA-LNPs were synthesized following the previously described method. After synthesis, the LNPs were diluted fivefold in PBS and purified by ultrafiltration at 10,000 × g. A549 cells were cultured in DMEM supplemented with 10% FBS and 1% P/S. Cells were seeded in 96-well plates at a density of 31,250 cells/cm^2^ and incubated at 37 °C with 5% CO_2_ 24 h. The culture medium was then replaced with 170 μL of serum-free DMEM and 30 μL of Cy3-siRNA-LNPs, corresponding to either 9 pmol (high) or 3 pmol (low) of Cy3-siRNA per well. After 6 h of incubation, the medium was replaced with PBS containing NucBlue™ Live ReadyProbes™ reagent and incubated for an additional 20 min. Fluorescence imaging was performed using an Olympus IX81 fluorescence microscope. The intracellular Cy3 signal was semi-quantitatively analyzed using ImageJ software.

### 2.5 In vitro gene silencing evaluation using S100P siRNA

To evaluate gene silencing efficiency, S100P-targeting siRNA was diluted in 25 mM sodium acetate buffer to a final concentration of 2 nmol/mL and encapsulated into LNPs using the MiNANO-form as described above. The resulting S100P-siRNA-LNPs were diluted fivefold and purified by ultrafiltration at 10,000 × g. A549 cells were cultured in DMEM supplemented with 10% FBS and 1% P/S. Cells were seeded into 12-well plates at a density of 31,250 cells/cm^2^ and incubated at 37 °C with 5% CO_2_ for 24 h. Subsequently, the medium was replaced with 750 μL of serum-free DMEM and 250 μL of S100P-siRNA-LNPs per well. After 6 h of incubation, the transfection medium was replaced with fresh complete medium. Cells were continuously cultured for an additional 42 h before RNA extraction. Total RNA was isolated using the RNeasy Mini Kit, and reverse transcription was performed using the SuperScript™ III First-Strand Synthesis SuperMix kit. Quantitative PCR (qPCR) was conducted using the TaqMan™ Universal PCR Master Mix on a QuantStudio™ 7 Flex System. S100P mRNA levels were quantified using a TaqMan gene expression assay (Hs00195584_m1), and GAPDH (Hs99999905_m1) served as the endogenous control. Relative gene expression was calculated using the comparative Ct (2^−ΔΔCt) method.

### 2.6 In vivo evaluation of selected LNP formulations: biodistribution and biosafety

Four LNP formulations that achieved the most effective in vitro gene silencing were selected for in vivo biodistribution analysis (Fig. 1c), which formulations were shown in Table. S. Cy3-labeled siRNA was diluted in 25 mM sodium acetate buffer (pH 5) to a final concentration of 40 nmol/mL and encapsulated into LNPs using the MiNANO-form platform. The resulting Cy3-siRNA-LNPs were purified by ultrafiltration at 10,000 × g. For each formulation, 150 μL of Cy3-siRNA-LNPs was intravenously injected into C57BL/6J mice (tail vein). Two hours post-injection, blood samples were first collected from the retro-orbital plexus and centrifuged to isolate serum. The serum levels of alanine aminotransferase (ALT), aspartate aminotransferase (AST), creatinine (CREA), urea (UREA), lactate dehydrogenase (LDH), and creatine kinase (CK) were measured using an automated biochemical analyzer to evaluate systemic biosafety. Following blood collection, the mice were euthanized, and major organs including the heart, liver, spleen, lungs, and kidneys were harvested. Organ-level fluorescence was visualized using an in vivo imaging system to assess the biodistribution of the Cy3-siRNA-LNPs.

### 2.7 Statistical and data analysis

The mean ± standard deviation (±SD) was presented for all data. For nanoparticle characterization (size, PDI, and zeta potential), each formulation was measured in triplicate within a single preparation to account for technical variation. No independent batch replicates were performed for these measurements. For cell-based experiments, each condition was tested in three wells per experiment. One-way ANOVA with Dunnett's multiple-comparison tests was used to analyzed the difference for different groups using Prism 8.0 (GraphPad). A difference was considered significant if P < 0.05 (*P < 0.05, **P < 0.01, ***P < 0.001, ****P < 0.0001).

## 3 Results

### 3.1 Physicochemical characterization of empty LNPs and correlation analysis

**Fig. 2:**
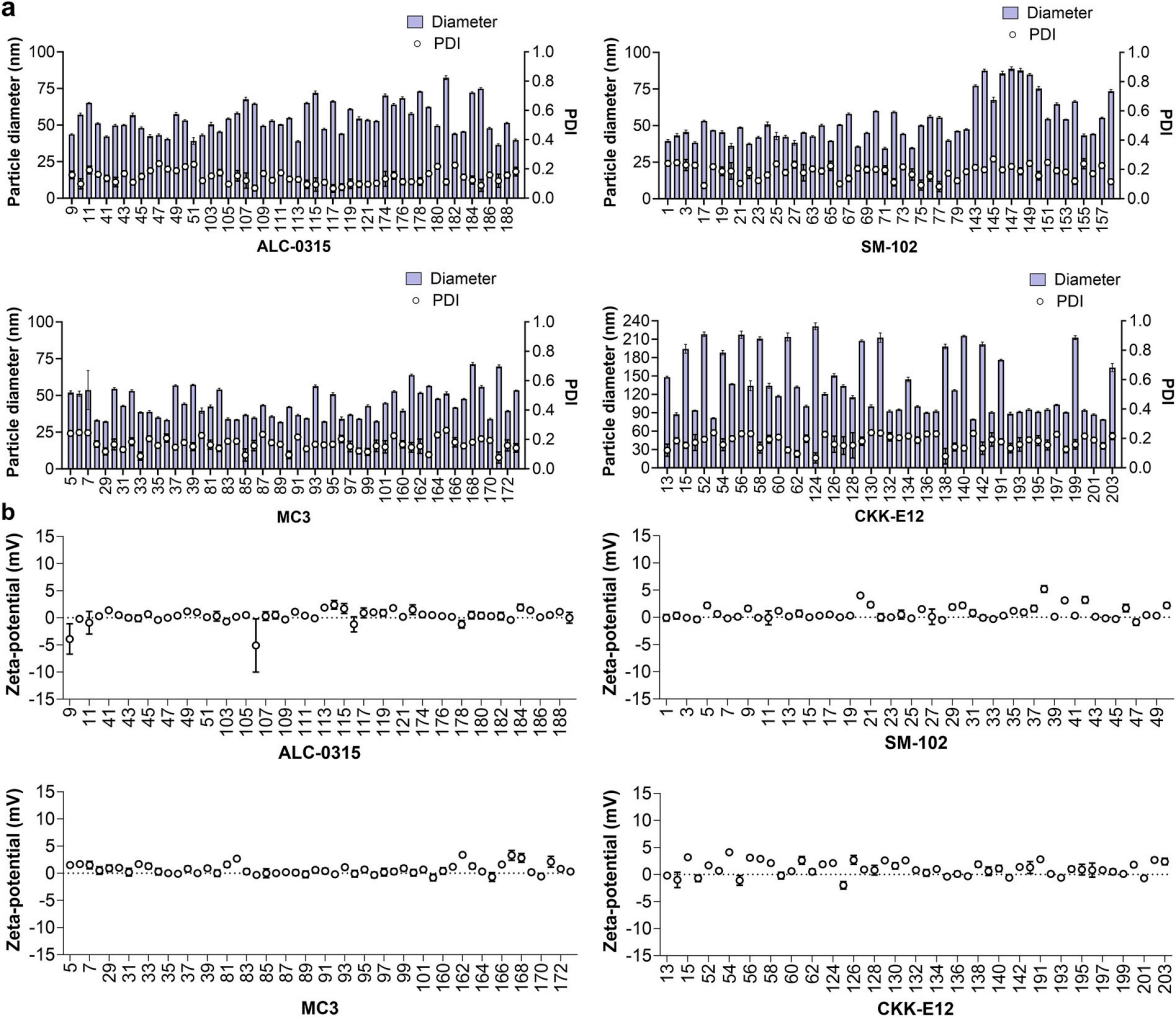
(a)The size and PDI of different ionizable lipids; (b) Zeta potential of different ionizable lipids.

To evaluate the physicochemical properties of the different LNP formulations, 203 representative candidates were selected from the pool of 864 designs and synthesized as empty LNPs using four ionizable lipids: ALC-0315, SM-102, MC3, and CKK-E12. The size and PDI of these formulations were shown in Fig. 2, Fig. S1, and S2. Both parameters varied depending on the ionizable lipid used. ALC-0315– and SM-102–based LNPs (Fig. 2a) exhibited relatively small sizes, mostly below 80 nm, and PDIs typically under 0.25, indicating uniform nanoparticle formation. MC3-based formulations (Fig. 2a) displayed the most consistent profiles, with sizes tightly clustered below 80 nm and PDIs centered around 0.15, reflecting excellent monodispersity. CKK-E12–containing formulations (Fig. 2a) showed a broader size distribution, with many particles ranging from 80 nm to 200 nm and some exceeding 200 nm; however, a substantial proportion of these formulations maintained PDIs around 0.15–0.20.

Zeta potential, another key physicochemical property, was also measured for all formulations (Fig. 2b). Although both the ionizable lipid type and the specific formulation composition influenced surface charge, all zeta potential values fell within a narrow range of −10 mV to +10 mV, with most formulations exhibiting a slightly positive charge. This range is generally considered favorable for cellular uptake, indicating that none of the formulations presented undesirable surface charge characteristics. Given the limited variability across formulations and the uniformly favorable zeta potential profiles, this parameter was not included as a feature in the subsequent machine learning model training.

**Fig. 3:**
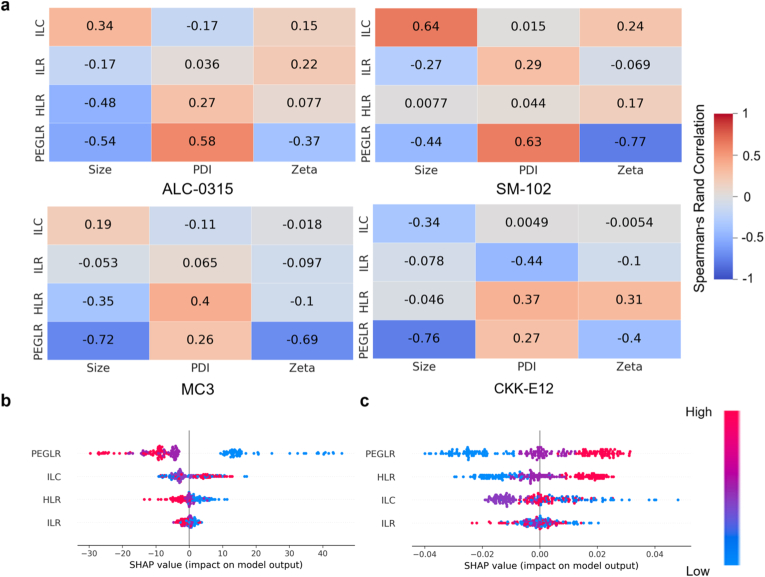
Analysis of relationships between formulation parameters and LNP properties. (a) Spearman's rank correlations between input features and output properties; (b) SHAP summary plot showing key contributors to size prediction; (c) SHAP summary plot showing feature effects for PDI prediction.

The relationships between formulation inputs (ionizable lipid concentration, ionizable lipid ratio, helper lipid ratio, and PEG-lipid ratio) and key outputs (size, PDI, and zeta potential) were evaluated using Spearman correlation analysis (Fig. 3a). Distinct correlation profiles were observed across the four ionizable lipids. Ionizable lipid concentration showed a positive correlation with size for ALC-0315, SM-102, and MC3, with SM-102 exhibiting the strongest association (ρ = 0.64), indicating higher size sensitivity to concentration changes. In contrast, CKK-E12 displayed a negative correlation between ionizable lipid concentration and size. The ionizable lipid ratio demonstrated generally weak associations with size, PDI, and zeta potential for ALC-0315, SM-102, and MC3, but for CKK-E12, a modest negative correlation with PDI was observed.

The helper lipid ratio showed a negative correlation with size and a positive correlation with PDI for ALC-0315 and MC3, while no strong associations were found for SM-102. For CKK-E12, the helper lipid ratio exhibited a weak positive correlation with PDI. Notably, across all four lipid types, the PEG-lipid ratio was consistently negatively correlated with size and positively correlated with PDI. These results underscore that formulation components influence LNP characteristics in a lipid-specific manner, highlighting the importance of tailoring optimization strategies to each ionizable lipid.

### 3.2 Machine learning–guided formulation screening based on LNP properties

#### 3.2.1 Algorithm performance for size and PDI prediction

To identify the most robust predictive models, we evaluated four regression algorithms: GBR, MLP, SVR, and LR as a baseline. The predictive performance of these models for size and PDI is summarized in Table 2, Table 3, respectively. In both tables, all reported metrics—R^2^, MAE, percent error, and Spearman's rank correlation coefficient (ρ)—are presented as the mean ± standard deviation calculated from 10 independent iterations.

**Table 2:** Model selection results for LNP transfection in size.

Model	R [2]	MAE (nm)	PE (%)	Spearman's ρ
GBR	0.87 ± 0.08	6.97 ± 1.49	9.64 ± 1.62	0.94 ± 0.03
MLP	0.80 ± 0.12	9.26 ± 1.39	12.97 ± 1.63	0.90 ± 0.04
SVR	0.87 ± 0.04	7.65 ± 1.34	10.31 ± 1.42	0.93 ± 0.018
LR	0.64 ± 0.23	14.90 ± 1.79	22.66 ± 2.67	0.82 ± 0.12

**Table 3:** Model selection results for LNP transfection in PDI.

Model	Spearman's ρ	PE (%)	MAE	R [2]
GBR	0.66 ± 0.05	20.41 ± 2.05	0.03 ± 0.00	0.40 ± 0.08
MLP	0.68 ± 0.07	20.53 ± 2.43	0.03 ± 0.00	0.36 ± 0.15
SVR	0.65 ± 0.07	21.25 ± 2.52	0.03 ± 0.00	0.35 ± 0.07
LR	0.59 ± 0.08	22.78 ± 2.33	0.03 ± 0.00	0.28 ± 0.14

For size (Table 2), all non-linear models achieved high R^2^ values (≥0.80), indicating good fitting capacity, whereas linear regression performed substantially worse (R^2^ = 0.64 ± 0.23). Among the non-linear models, GBR and SVR showed similarly high R^2^ (both 0.87), but GBR achieved the lowest MAE (6.97 ± 1.49 nm) and percent error (9.64 ± 1.62%) together with the highest rank correlation (Spearman's ρ = 0.94 ± 0.03). These results indicate that GBR not only explains the largest proportion of variance in size, but also provides the most accurate and monotonic mapping between predicted and observed values. On this basis, GBR was selected as the primary model for size prediction.

For PDI, the models showed weaker performance in terms of R^2^ (Table 3). Across GBR, MLP and SVR, R^2^ values were modest (0.35–0.40) and percent error remained around 20%, with similar MAE values (∼0.03), but all three non-linear models outperformed the linear baseline, which showed lower R^2^ and Spearman's ρ and higher PE. For PDI, however, the main objective is not only to match the exact numerical value, but to preserve a correct ordering of formulations, because smaller PDI indicates more uniform and therefore more desirable particles. The moderate rank correlations (Spearman's ρ ≈ 0.65–0.68) show that the models capture this monotonic relationship and can rank formulations reasonably well according to their polydispersity. This is valuable for screening even when R^2^ is limited. The relatively modest R^2^ values are consistent with the nature of the PDI endpoint: the target distribution is highly skewed and imbalanced (Fig. S1–S2), and PDI is sensitive to process-related variability (e.g. transient aggregation, equipment noise and measurement fluctuations [36]) as well as formulation composition. In addition, key process variables such as temperature and preparation time were not recorded, which naturally constrains the maximal achievable performance of any data-driven model. GBR was selected for subsequent formulation screening. GBR provided a good balance between rank preservation and point-wise accuracy: its Spearman's ρ (0.66 ± 0.05) was close to the highest value observed, while it achieved the best R^2^ (0.40 ± 0.08) and the lowest percent error (20.41 ± 2.05%) among all models, with MAE remaining low (∼0.03). In other words, GBR reliably distinguishes formulations with lower (more favorable) PDI from those with higher PDI, while at the same time offering slightly more accurate numerical predictions than the alternatives. Thus, despite the limited R^2^ inherent to the PDI endpoint, the GBR model is sufficiently informative for prioritising formulations based on PDI and is suitable for use in the comparative screening workflow.

Because the PDI model showed more modest performance than the size model, additional diagnostics were performed to better characterize its reliability and limitations. The predicted-versus-observed and residual plots showed that the final PDI gradient boosting regressor captured the overall PDI trend but with moderate prediction error, supporting its use as an approximate ranking tool rather than a precise predictor (Fig. S3–S5). Stratified error analysis showed broadly comparable absolute errors across ionizable lipid types, with slightly higher variability for CKK-E12 (Fig. S6). Y-randomization controls based on R^2^ and Spearman's ρ showed that the real model clearly outperformed randomized-label models, suggesting that the observed PDI prediction and ranking performance was unlikely to arise from chance correlations alone (Fig. S7–S8).

A post hoc bootstrap-based uncertainty analysis was further performed for the final PDI model. The bootstrap upper 95% bounds increased with the mean predicted PDI values, while the 95% interval widths varied across samples (Fig. S9–S10). In addition, samples with similar predicted PDI values showed different uncertainty levels (Fig. S11), indicating that point predictions alone do not fully reflect model reliability. Therefore, the PDI model was used primarily as a tool for approximate ranking and prioritization, rather than as a precise predictor of PDI values.

#### 3.2.2 Feature impact analysis on LNP size and PDI

SHAP (SHapley Additive exPlanations) analysis was applied to interpret the contribution of each input feature to the outputs of the trained models (Fig. 3b and c). In these summary plots, each point represents a single sample, with its position on the x-axis indicating the SHAP value, how strongly that feature influenced the model output for that sample. Positive values indicate a positive contribution to the prediction, while negative values indicate a suppressive effect. The color gradient reflects the magnitude of the feature value (blue for low, red for high), and the vertical axis ranks features by their overall importance.

For size prediction (Fig. 3b), PEGLR (PEG-lipid ratio) and ILC (ionizable lipid concentration) were the top contributors, highlighting the central role of both the relative and absolute proportions of ionizable and PEG lipids in governing particle size. In contrast, the PDI prediction model (Fig. 3c) showed SHAP values that were more scattered and centered closer to zero, indicating weaker and less stable predictive signals. This aligns with the model's modest performance for PDI prediction and suggests the absence of a dominant feature driving PDI across the dataset. Nevertheless, subtle patterns, such as localized SHAP shifts for PEGLR and HLR, hint at potential secondary effects that could be explored in future data collection or through refined feature engineering.

#### 3.2.3 ML-guided identification of candidate LNP formulations

The trained machine learning models were used to screen the 864 candidate formulations generated from combinatorial variations of ionizable lipid type, helper lipid type, molar ratio, and lipid concentration. For each candidate, the predicted size and PDI values were independently normalized to a [0,1] scale before ranking, so that the two outputs could be compared within the same scoring framework. A composite score was then calculated by assigning a weight of 0.7 to normalized size and 0.3 to normalized PDI, with lower scores indicating more favorable predicted formulation profiles. The weighting was empirically defined, with a larger contribution assigned to size because the size model showed stronger predictive performance and because particle size was the primary physicochemical property considered at this screening stage. PDI was included as a secondary criterion to penalize formulations with relatively high dispersity. Given the more modest predictive accuracy of the PDI model, its use in this score was intended to provide relative prioritization rather than precise quantitative prediction.

Candidate formulations were ranked within each ionizable lipid group according to the composite score. A total of 25 formulations were selected for in vitro validation, including seven ALC-0315 formulations and six formulations from each of the other ionizable lipid groups. The selected formulations are listed in Table S2, together with their composition, predicted size, predicted PDI, experimentally measured size and PDI, cellular uptake, and gene silencing efficiency. This screening strategy was used to reduce the full candidate space to a manageable set of formulations for downstream biological evaluation while retaining candidates with small predicted size and relatively low predicted dispersity. The Python code used in this study is available at https://github.com/luhan611/siRNA-LNP-ML-Optimization.git.

### 3.3 Cellular uptake of optimized Cy3-siRNA LNP formulations

**Fig. 4:**
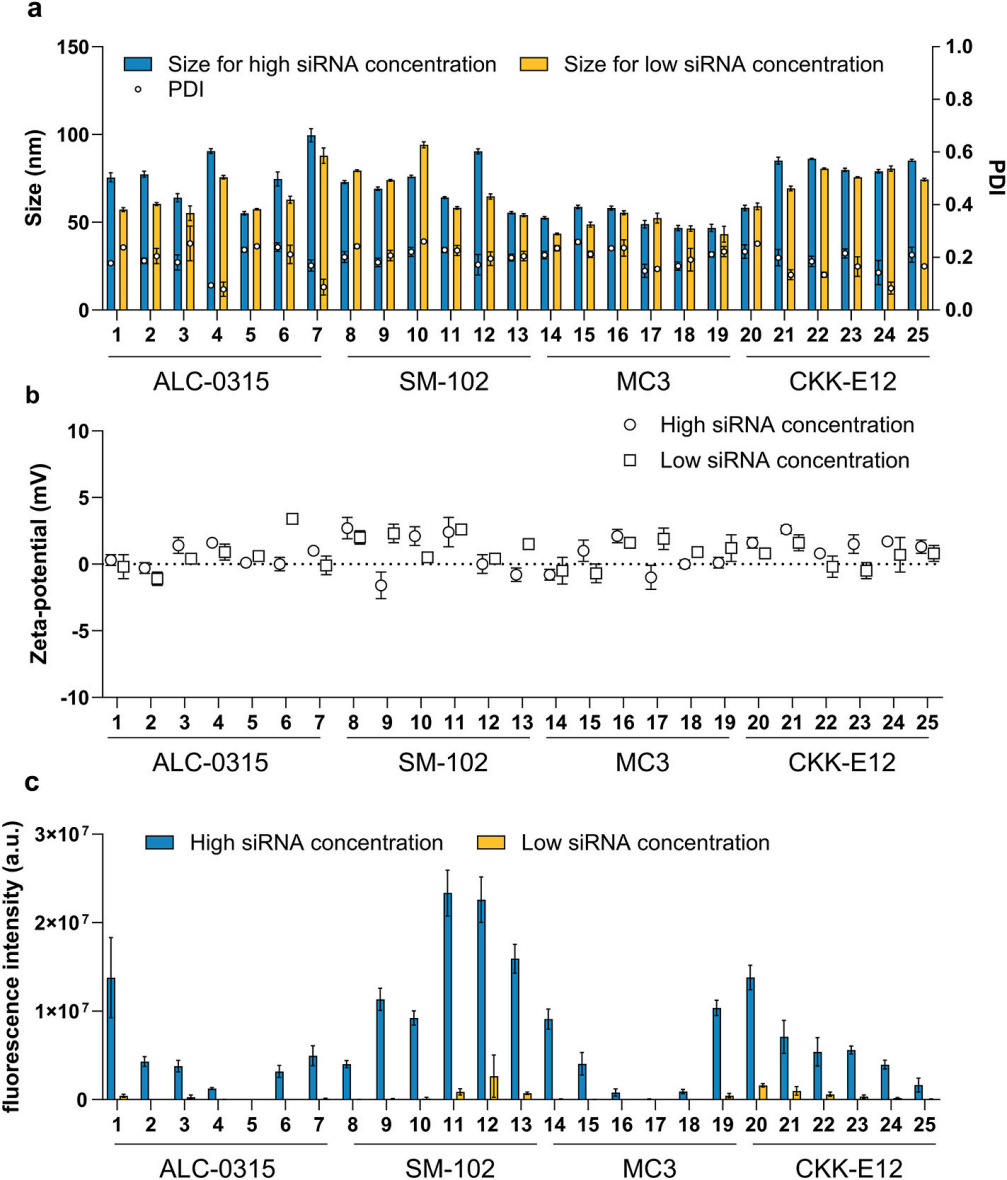
Characterization of 25 LNP formulations loaded with Cy3-labeled siRNA. (a) Size and PDI of LNPs; (b) Zeta potential of LNPs; (c) Cellular uptake efficiency in A549 cells, quantified by fluorescence intensity.

The size, PDI, and zeta potential of the top 25 LNP formulations encapsulating varying concentrations of Cy3-labeled siRNA were shown in Fig. 4a and b. Following siRNA encapsulation, the size of ALC-0315, SM-102, and MC3 LNPs slightly increased, while the size of CKK-E12 LNPs showed a modest decrease. Notably, the size of all formulations remained below 100 nm. The PDI values of several formulations exceeded 0.2 but remained below 0.25 across all samples, indicating acceptable particle uniformity. Higher siRNA loading concentrations resulted in larger sizes, consistent with increased siRNA encapsulation within the nanoparticles. The extent of size increased relative to the predicted size varied across different formulations, reflecting differences in formulation composition. The zeta potential of all groups ranged from −2 mV to +3 mV (Fig. 4b), suggesting a near-neutral surface charge for all formulations.

The cellular uptake efficiency of the Cy3-siRNA-loaded LNPs was presented in Fig. 4c and Fig. S12. Distinct uptake efficiencies were observed across different formulations, with higher siRNA loading concentrations resulting in significantly enhanced uptake compared to lower concentrations. Among the tested formulations, SM-102-based LNPs exhibited the highest uptake efficiency, followed by ALC-0315 and CKK-E12 LNPs. In contrast, MC3-based LNPs demonstrated the lowest uptake efficiency under the tested conditions.

### 3.4 S100P-siRNA delivery and in vitro gene silencing efficacy

**Fig. 5:**
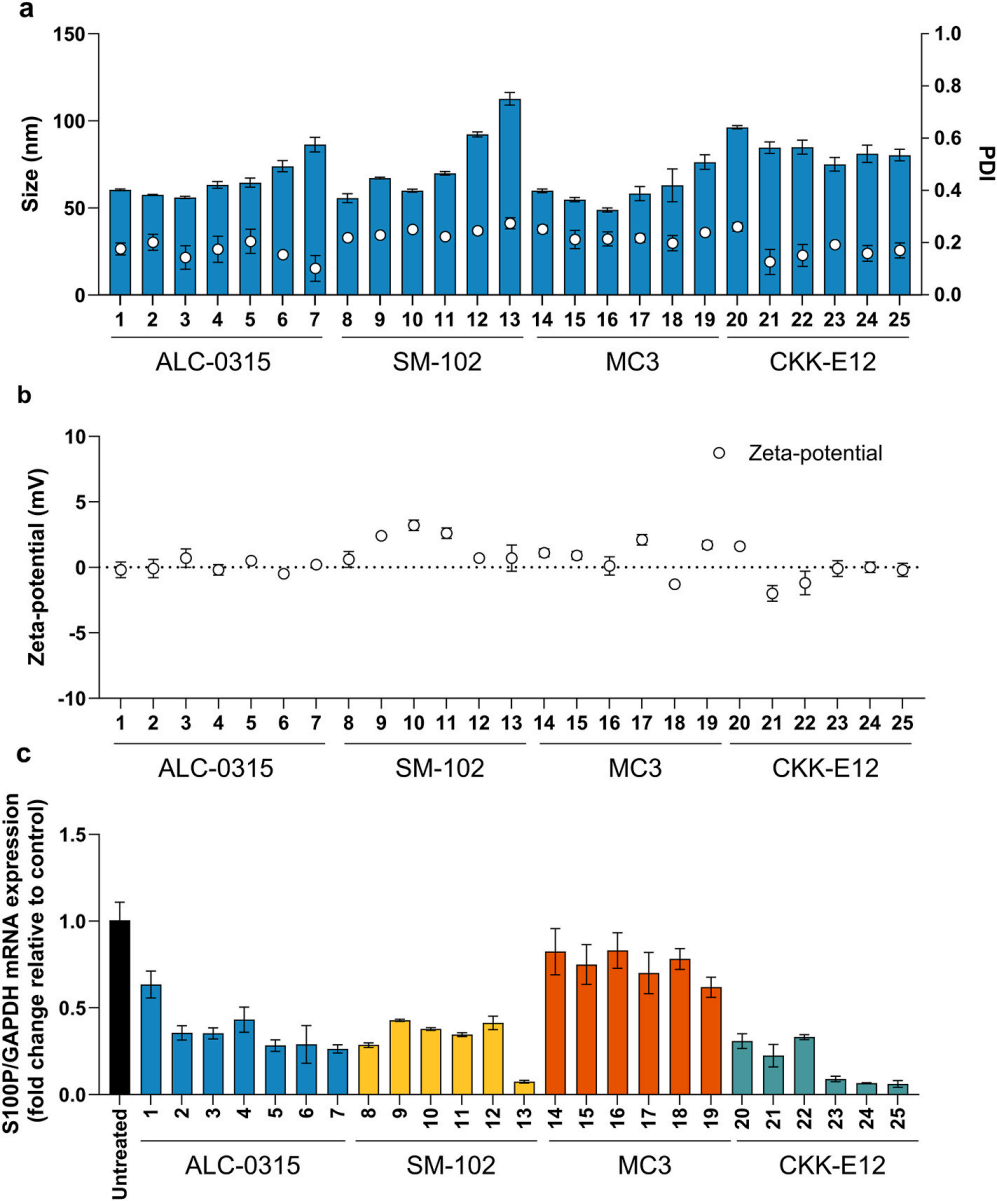
Characterization and gene silencing performance of 25 LNP formulations loaded with S100P siRNA. (a) Size and PDI of LNPs; (b) Zeta potential of LNPs. (c) Gene silencing efficiency in A549 cells, measured by S100P mRNA expression relative to untreated control.

To evaluate gene silencing efficiency, S100P siRNA was used as a therapeutic target. Based on the previous observation that higher siRNA concentrations yielded superior cellular uptake, a high siRNA concentration was employed to assess S100P mRNA expression levels. The size, PDI, and zeta potential of the resulting S100P-siRNA-loaded LNPs were presented in Fig. 5a and b. Sizes ranged from approximately 55 to 112 nm across formulations. ALC-0315- and MC3-based LNPs generally exhibited smaller sizes (50–80 nm), whereas SM-102- and CKK-E12-based LNPs displayed larger sizes. All formulations maintained PDI values below 0.25, indicative of good particle uniformity. Compared to the corresponding Cy3-siRNA-loaded LNPs, similar size trends were observed, although slight variations across certain formulations may reflect the influence of siRNA sequence and vendor-specific chemical modifications. The zeta potential of all formulations ranged from −2 mV to +3 mV (Fig. 5b), indicating near-neutral surface charge.

The gene silencing performance of the different formulations was assessed by measuring S100P mRNA expression levels (Fig. 5c). CKK-E12-based LNPs demonstrated the strongest silencing effect, with formulations 24 and 25 achieving up to 92% knockdown efficiency. Several ALC-0315- and SM-102-based LNPs also exhibited robust gene silencing; notably, formulation 13 (SM-102) achieved ∼90% knockdown, while MC3-based LNPs consistently produced high knockdown efficiencies. These results underscore the capacity of selected formulations to effectively deliver S100P siRNA and induce target gene suppression.

To identify candidates for in vivo biodistribution studies, four representative formulations were selected based on a combined assessment of S100P mRNA knockdown efficiency and cellular uptake performance. For ALC-0315, formulation 7 was chosen for its balanced profile, showing moderate knockdown efficiency alongside favorable uptake. For SM-102, formulation 13 demonstrated the highest knockdown efficiency, even though its uptake efficiency was slightly lower, warranting its inclusion. For MC3, formulation 19 achieved the highest performance in both uptake and knockdown, making it the optimal representative for this lipid type. For CKK-E12, both formulations 24 and 25 showed strong knockdown activity; formulation 24, which also exhibited superior uptake, was prioritized for further studies. Based on these in vitro findings, the four selected formulations were advanced to in vivo biodistribution experiments to evaluate their delivery efficiency and organ targeting potential.

### 3.5 In vivo biodistribution of selected LNP formulations

**Fig. 6:**
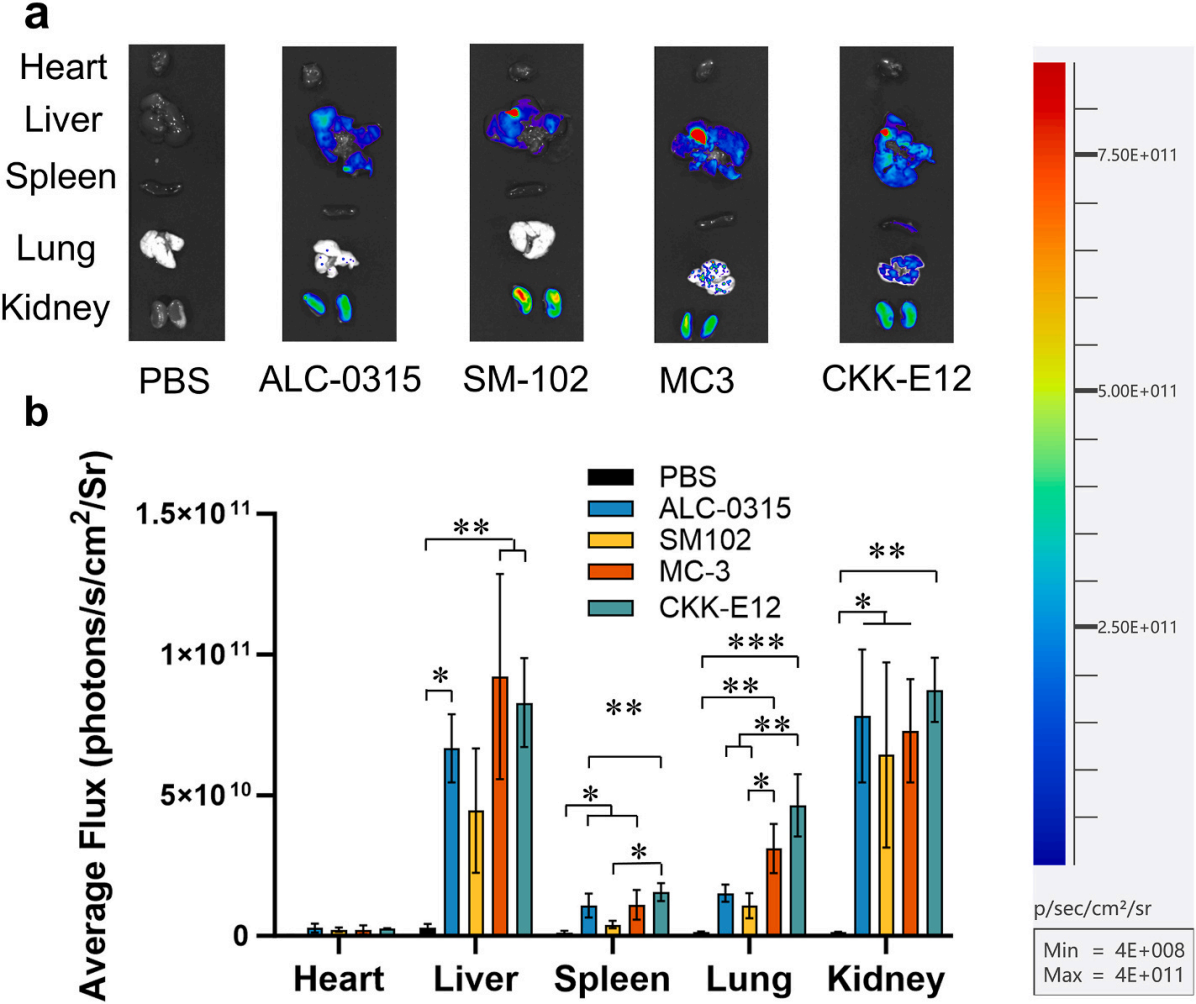
In vivo biodistribution of Cy3-labeled siRNA-loaded LNPs in C57BL/6J mice. (a) Representative ex vivo fluorescence images of major organs (heart, liver, spleen, lung, kidney) at 2 h post-intravenous injection of LNPs formulated with different ionizable lipids. (b) Quantitative analysis of average fluorescence intensity in each organ.

To assess the in vivo biodistribution of the selected LNP formulations (shown in Table S3), Cy3-labeled siRNA-loaded LNPs were intravenously administered to mice via the tail vein. Two hours post-injection, major organs (heart, liver, spleen, lungs, and kidneys) were harvested for imaging using IVIS. Representative fluorescence images (Fig. 6a) revealed strong signals in the liver and kidneys for all formulations, consistent with typical clearance pathways of lipid nanoparticles. Notably, CKK-E12-based LNPs showed the highest fluorescence intensity in the lungs, while MC3-based LNPs exhibited moderate lung accumulation. By contrast, ALC-0315- and SM-102-based formulations demonstrated lower lung uptake but strong liver and kidney accumulation. Quantitative analysis of average fluorescence intensity (Fig. 6b) confirmed these trends. Lung fluorescence intensity was significantly higher for CKK-E12-based LNPs and moderately elevated for MC3-based LNPs compared with ALC-0315 and SM-102. In the spleen, CKK-E12 and MC3 formulations yielded stronger signals than SM-102 (p < 0.05), whereas heart accumulation remained minimal across all groups. Together, these results demonstrate that ionizable lipid composition strongly influences organ-level biodistribution. The enhanced pulmonary tropism of CKK-E12-based LNPs highlights their potential for targeted lung delivery in siRNA therapeutic applications.

### 3.6 In vivo biosafety assessment of LNP formulations

**Fig. 7:**
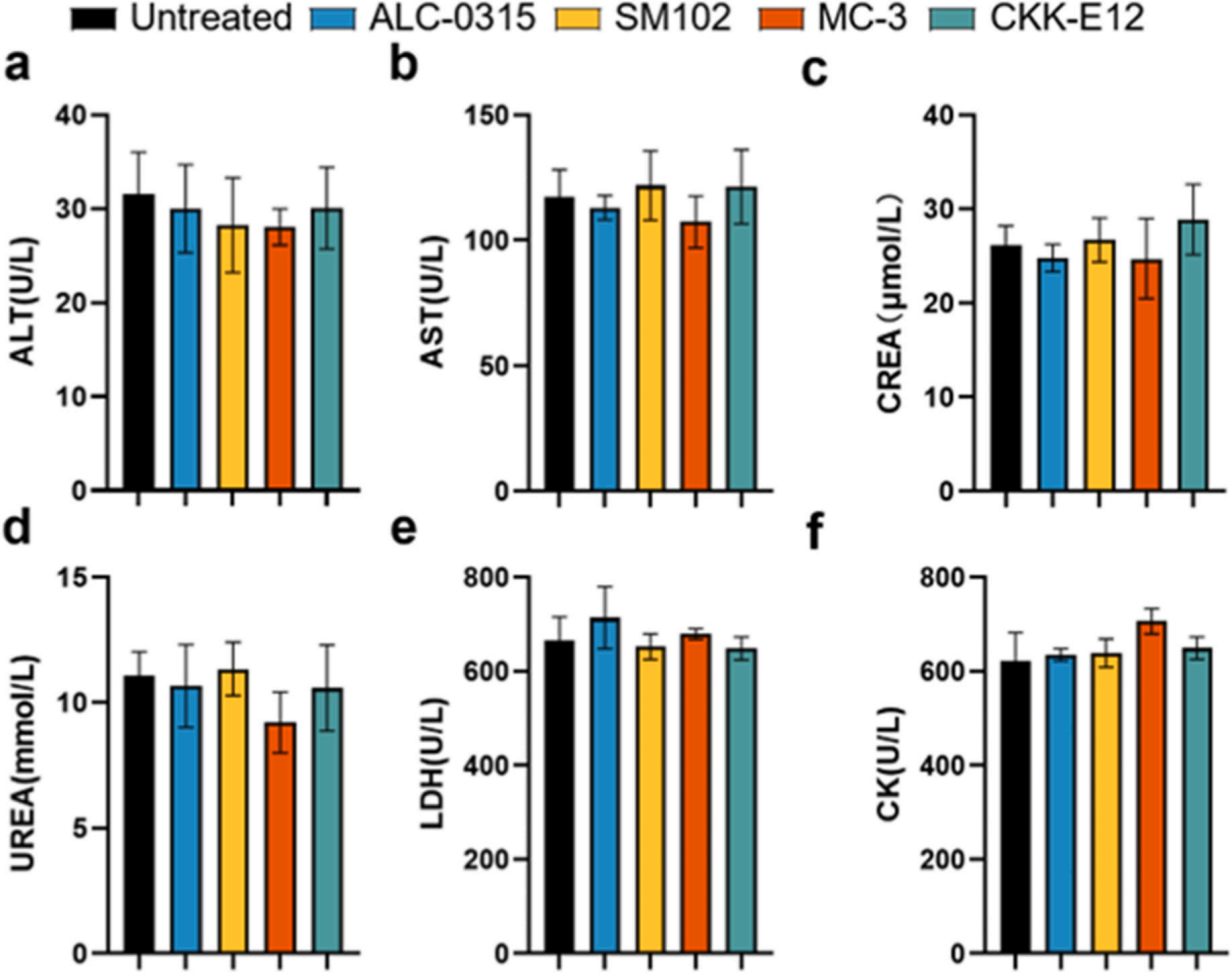
Analysis of biochemical results in mice. (a) Statistical analysis of ALT index detection in mouse serum. (b) Statistical analysis of AST index detection in mouse serum. (c) Statistical analysis of CREA index detection in mouse serum. (d) Statistical analysis of UREA index detection in mouse serum. (e) Statistical analysis of LDH index detection in mouse serum. (f) Statistical analysis of CK index detection in mouse serum.

In order to further investigate the in vivo biosafety of the selected LNP formulations, blood samples were collected from mice injected with different LNP preparations via the tail vein for blood biochemical index detection. The serum liver function (ALT, AST), kidney function (CREA, UREA), and myocardial enzyme (LDH, CK) biomarkers of the mice were maintained within the normal reference range (Fig. 7a–f), indicating that different LNP formulations have good biosafety and provide biosafety guarantees for further in vivo treatment.

## 4 Discussion

In this study, we established a high-throughput microfluidic and ML-assisted workflow for the stepwise screening and prioritization of LNP formulations for siRNA delivery. The overall screening process was designed as a stepwise strategy. A library containing 864 candidate LNP formulations was first generated based on different ionizable lipids, helper lipids, lipid molar ratios, and lipid concentrations. From this full library, 203 formulations were randomly selected, experimentally prepared as empty LNPs, and characterized in terms of particle size, PDI, and zeta potential. These 203 experimentally tested formulations represented a subset of the 864-formulation library and were used to train regression models for physicochemical prediction. After model training, particle size and PDI were evaluated across the full 864-formulation library, including both the experimentally characterized formulations and the untested model-predicted formulations. Based on a composite ranking criterion incorporating particle size and PDI, 25 candidate formulations were selected for siRNA loading and biological validation.

This workflow reduces the experimental burden associated with large-scale LNP formulation screening. Direct preparation and biological testing of all 864 candidate formulations would require substantially greater amounts of lipids, siRNA, cell culture reagents, qPCR materials, and experimental time. In contrast, the present strategy required experimental physicochemical characterization of only 203 formulations, corresponding to approximately 23.5% of the full library. This allowed the more costly and time-consuming biological assays to be focused on a smaller number of prioritized candidates, while still maintaining broad coverage of the formulation design space through model-assisted prediction.

The in vitro results further demonstrated the necessity of biological validation after physicochemical prescreening. Although small particle size and low PDI are generally desirable for LNP formulation development, these parameters alone did not fully determine cellular uptake or gene silencing efficiency. Some formulations with favorable predicted or measured physicochemical properties did not show the highest biological activity, indicating that delivery performance depends on additional formulation- and payload-related factors. That means internal structures affect affect cellular uptake, gene silencing efficiency, and biodistribution [37]. In the Cy3-siRNA uptake assay, the lower fluorescence intensity observed at the lower siRNA dose may be partly attributed to the reduced amount of fluorescently labeled siRNA available for cellular internalization and microscopic detection. Therefore, microscopic fluorescence intensity should be interpreted in the context of siRNA dose, formulation composition, and downstream functional assays. In addition, differences between Cy3-labeled siRNA-loaded LNPs and S100P siRNA-loaded LNPs may arise from differences in siRNA sequence, length, labelling, and payload–lipid interactions.

Several limitations should be acknowledged. First, this study mainly focused on formulation composition, whereas additional process parameters, such as total flow rate, flow rate ratio, buffer composition, and siRNA concentration, may also affect LNP properties and delivery performance. These factors should be included in future optimization. Second, because the PDI model showed only moderate predictive performance, PDI may be better used as a practical screening criterion rather than a precise prediction target. For example, formulations with PDI values higher than 0.2 could be excluded, while formulations with PDI values below 0.2 could be considered for further testing. Third, the current in vivo study focused on organ distribution and biosafety, and future work should evaluate in vivo gene silencing efficiency using disease-relevant siRNA to further confirm the therapeutic potential of the selected formulations.

## 5 Conclusion

In conclusion, this study developed a high-throughput microfluidic and ML-assisted workflow for LNP formulation screening. Using 203 experimentally characterized empty LNPs from a library of 864 candidates, the particle size and PDI of the entire library were predicted, and formulations were prioritized based on their physicochemical properties, resulting in 25 candidates for siRNA loading and biological validation. This approach reduced experimental burden while preserving broad formulation-space coverage. The machine learning component served as a physicochemical prescreening tool, and biological performance was confirmed experimentally. Overall, this workflow provides a practical strategy for integrating high-throughput preparation, model-assisted prioritization, and biological validation in future siRNA-LNP development.

## CRediT authorship contribution statement

**Mingzhi Yu:** Conceptualization, Data curation, Methodology, Writing – original draft. **Luhan Wang:** Conceptualization, Methodology, Software, Writing – original draft. **Dongsheng Liu:** Conceptualization, Methodology. **Jianqiang Guo:** Data curation, Methodology, Writing – original draft. **Jiahao Liu:** Data curation, Methodology. **Jiawei Zhou:** Data curation, Methodology. **Allen Mathew:** Methodology. **Zhonglei He:** Funding acquisition, Supervision, Writing – review & editing. **Jing Wu:** Funding acquisition, Supervision, Writing – review & editing. **Nan Zhang:** Conceptualization, Project administration, Supervision, Writing – review & editing.

## Declaration of competing interest

The authors declare the following financial interests/personal relationships which may be considered as potential competing interests: Nan Zhang, Jing Wu, Zhonglei He and Mingzhi Yu reports financial support was provided by Research Ireland, Enterprise Ireland, Open Research Fund of Key Lab of Industrial Dust Deep Reduction and Occupational Health and Safety of Anhui Higher Education Institutes, the Scientific Research Foundation for Talents of AUST and CSC. Nan Zhang, Dongsheng Liu, Mingzhi Yu and Allen Mathew has patent issued to GB2303464.8. If there are other authors, they declare that they have no known competing financial interests or personal relationships that could have appeared to influence the work reported in this paper.

## Data Availability

Data will be made available on request.
