# Supplementary material for: High-throughput microfluidics and machine learning-assisted screening of lipid nanoparticle formulations for siRNA delivery
Source: Mater Today Bio. 2026 Jul 22;39:103483. doi: 10.1016/j.mtbio.2026.103483 (PMC13450591; doi:10.1016/j.mtbio.2026.103483)
Supplement: Supplementary file 1 [file mmc1.docx]

Supplementary Material

**Machine learning enabled high-throughput microfluidic based screening and optimization of lipid nanoparticles for siRNA delivery**

*Mingzhi Yu^1^, Luhan Wang^1^, Dongsheng Liu^2, 3^, Jianqiang Guo^4^, Jiahao Liu^6^, Jiawei Zhou^4^, Allen Mathew^1^, Zhonglei He^6, 7*^, Jing Wu^4,5*^, Nan Zhang^1*^*

1. Centre of Micro/Nano Manufacturing Technology (MNMT-Dublin), School of Mechanical & Materials Engineering, University College Dublin, Dublin 4, D04 V1W8 Ireland.

2. Department of Aerospace and Mechanical Engineering, South East Technological University, Carlow, R93 N9R3, Ireland.

3. The Centre for Research and Enterprise in Engineering (engCORE), South East Technological University, Carlow, R93 N9R3, Ireland.

4. School of Medicine, Anhui University of Science and Technology, Huainan, 232001, China.

5 Key Laboratory of Industrial Dust Deep Reduction and Occupational Health and Safety of Anhui Higher Education Institutes, AUST, Huainan, 232001, China.

6. Institute of Precision Medicine (AUST-IPM), Anhui University of Science and Technology, Huainan 232001, China.

7. School of Public Health, Anhui University of Science and Technology, Huainan 232001, China.

^*^Corresponding author: [nan.zhang@ucd.ie](mailto:nan.zhang@ucd.ie); aust_jingwu@aust.edu.cn; hzlph@aust.edu.cn.

Fig. S1 Comparison of size distribution across different ionizable lipids

Fig. S2 Comparison of PDI distribution across different ionizable lipids

Fig. S3 Predicted versus observed PDI for the final gradient boosting regressor.

Fig. S4 Residual plot for the final PDI gradient boosting regressor.

Fig. S5 Residual distribution for the final PDI gradient boosting regressor.

Fig. S6 Absolute PDI error stratified by ionizable lipid type.

Fig. S7 Y-randomization control for the PDI model based on R².

Fig. S8 Y-randomization control for the PDI model based on Spearman’s rank correlation.

Fig. S9 Predicted PDI versus bootstrap 95% interval width.

Fig. S10 Distribution of bootstrap 95% interval widths for PDI prediction.

Fig. S11 Bootstrap mean predicted PDI versus upper 95% bound.

Figure S12 Uptake results of different formulations. (a) High siRNA concentration; (b) Low siRNA concentration.

Table S1. Selected 203 LNP formulations using Latin Hypercube Sampling

| No | Ionizable Lipid Type | Helper Lipid Type | Ionizable Lipid Concentration (mM) | Ionizable Lipid Ratio (%) | Helper Lipid Ratio (%) | PEG Lipid Ratio (%) |
| --- | --- | --- | --- | --- | --- | --- |
| 1 | SM-102 | DSPC | 1.25 | 50 | 16 | 2.5 |
| 2 | SM-102 | DSPC | 1.25 | 60 | 4 | 1.5 |
| 3 | SM-102 | DOPC | 1.25 | 60 | 4 | 1.5 |
| 4 | SM-102 | DOPC | 1.25 | 50 | 4 | 2.5 |
| 5 | MC-3 | DSPC | 1.25 | 40 | 16 | 0.5 |
| 6 | MC-3 | DSPC | 1.25 | 40 | 16 | 1.5 |
| 7 | MC-3 | DOPC | 1.25 | 50 | 10 | 1.5 |
| 8 | MC-3 | DOPC | 1.25 | 40 | 4 | 2.5 |
| 9 | ALC-0315 | DSPC | 1.25 | 40 | 4 | 2.5 |
| 10 | ALC-0315 | DSPC | 1.25 | 40 | 10 | 0.5 |
| 11 | ALC-0315 | DOPC | 1.25 | 40 | 4 | 0.5 |
| 12 | ALC-0315 | DOPC | 1.25 | 40 | 4 | 2.5 |
| 13 | CKK-E12 | DSPC | 1.25 | 60 | 10 | 0.5 |
| 14 | CKK-E12 | DSPC | 1.25 | 50 | 4 | 2.5 |
| 15 | CKK-E12 | DOPC | 1.25 | 40 | 10 | 0.5 |
| 16 | CKK-E12 | DOPC | 1.25 | 60 | 10 | 2.5 |
| 17 | SM-102 | DSPC | 2.5 | 40 | 10 | 0.5 |
| 18 | SM-102 | DSPC | 2.5 | 50 | 4 | 1.5 |
| 19 | SM-102 | DSPC | 2.5 | 40 | 10 | 2.5 |
| 20 | SM-102 | DSPC | 2.5 | 60 | 4 | 0.5 |
| 21 | SM-102 | DSPC | 2.5 | 40 | 16 | 0.5 |
| 22 | SM-102 | DSPC | 2.5 | 50 | 4 | 2.5 |
| 23 | SM-102 | DOPC | 2.5 | 40 | 4 | 1.5 |
| 24 | SM-102 | DOPC | 2.5 | 40 | 16 | 0.5 |
| 25 | SM-102 | DOPC | 2.5 | 60 | 16 | 2.5 |
| 26 | SM-102 | DOPC | 2.5 | 40 | 10 | 0.5 |
| 27 | SM-102 | DOPC | 2.5 | 50 | 10 | 2.5 |
| 28 | SM-102 | DOPC | 2.5 | 60 | 16 | 0.5 |
| 29 | MC-3 | DSPC | 2.5 | 60 | 4 | 2.5 |
| 30 | MC-3 | DSPC | 2.5 | 50 | 4 | 0.5 |
| 31 | MC-3 | DSPC | 2.5 | 40 | 4 | 1.5 |
| 32 | MC-3 | DSPC | 2.5 | 40 | 10 | 0.5 |
| 33 | MC-3 | DSPC | 2.5 | 60 | 4 | 1.5 |
| 34 | MC-3 | DSPC | 2.5 | 60 | 10 | 1.5 |
| 35 | MC-3 | DOPC | 2.5 | 40 | 16 | 1.5 |
| 36 | MC-3 | DOPC | 2.5 | 40 | 16 | 2.5 |
| 37 | MC-3 | DOPC | 2.5 | 50 | 4 | 0.5 |
| 38 | MC-3 | DOPC | 2.5 | 60 | 10 | 0.5 |
| 39 | MC-3 | DOPC | 2.5 | 60 | 4 | 0.5 |
| 40 | MC-3 | DOPC | 2.5 | 50 | 16 | 1.5 |
| 41 | ALC-0315 | DSPC | 2.5 | 60 | 16 | 1.5 |
| 42 | ALC-0315 | DSPC | 2.5 | 50 | 16 | 0.5 |
| 43 | ALC-0315 | DSPC | 2.5 | 40 | 16 | 1.5 |
| 44 | ALC-0315 | DSPC | 2.5 | 50 | 10 | 0.5 |
| 45 | ALC-0315 | DSPC | 2.5 | 40 | 4 | 1.5 |
| 46 | ALC-0315 | DOPC | 2.5 | 50 | 10 | 2.5 |
| 47 | ALC-0315 | DOPC | 2.5 | 50 | 16 | 2.5 |
| 48 | ALC-0315 | DOPC | 2.5 | 40 | 16 | 1.5 |
| 49 | ALC-0315 | DOPC | 2.5 | 50 | 16 | 0.5 |
| 50 | ALC-0315 | DOPC | 2.5 | 50 | 10 | 1.5 |
| 51 | ALC-0315 | DOPC | 2.5 | 60 | 10 | 2.5 |
| 52 | CKK-E12 | DSPC | 2.5 | 40 | 4 | 0.5 |
| 53 | CKK-E12 | DSPC | 2.5 | 60 | 16 | 2.5 |
| 54 | CKK-E12 | DSPC | 2.5 | 60 | 16 | 0.5 |
| 55 | CKK-E12 | DSPC | 2.5 | 40 | 4 | 1.5 |
| 56 | CKK-E12 | DSPC | 2.5 | 40 | 16 | 0.5 |
| 57 | CKK-E12 | DOPC | 2.5 | 40 | 16 | 1.5 |
| 58 | CKK-E12 | DOPC | 2.5 | 40 | 10 | 0.5 |
| 59 | CKK-E12 | DOPC | 2.5 | 50 | 10 | 1.5 |
| 60 | CKK-E12 | DOPC | 2.5 | 50 | 16 | 2.5 |
| 61 | CKK-E12 | DOPC | 2.5 | 50 | 10 | 0.5 |
| 62 | CKK-E12 | DOPC | 2.5 | 60 | 4 | 1.5 |
| 63 | SM-102 | DSPC | 5 | 60 | 10 | 1.5 |
| 64 | SM-102 | DSPC | 5 | 40 | 10 | 1.5 |
| 65 | SM-102 | DSPC | 5 | 60 | 16 | 2.5 |
| 66 | SM-102 | DSPC | 5 | 50 | 16 | 0.5 |
| 67 | SM-102 | DSPC | 5 | 40 | 4 | 0.5 |
| 68 | SM-102 | DSPC | 5 | 60 | 10 | 2.5 |
| 69 | SM-102 | DSPC | 5 | 50 | 4 | 2.5 |
| 70 | SM-102 | DSPC | 5 | 40 | 4 | 1.5 |
| 71 | SM-102 | DOPC | 5 | 60 | 16 | 2.5 |
| 72 | SM-102 | DOPC | 5 | 40 | 10 | 0.5 |
| 73 | SM-102 | DOPC | 5 | 60 | 4 | 1.5 |
| 74 | SM-102 | DOPC | 5 | 40 | 16 | 2.5 |
| 75 | SM-102 | DOPC | 5 | 50 | 10 | 0.5 |
| 76 | SM-102 | DOPC | 5 | 50 | 16 | 0.5 |
| 77 | SM-102 | DOPC | 5 | 60 | 10 | 0.5 |
| 78 | SM-102 | DOPC | 5 | 40 | 4 | 2.5 |
| 79 | SM-102 | DOPC | 5 | 40 | 4 | 1.5 |
| 80 | SM-102 | DOPC | 5 | 50 | 4 | 1.5 |
| 81 | MC-3 | DSPC | 5 | 60 | 16 | 0.5 |
| 82 | MC-3 | DSPC | 5 | 40 | 10 | 0.5 |
| 83 | MC-3 | DSPC | 5 | 60 | 16 | 1.5 |
| 84 | MC-3 | DSPC | 5 | 50 | 16 | 2.5 |
| 85 | MC-3 | DSPC | 5 | 50 | 10 | 1.5 |
| 86 | MC-3 | DSPC | 5 | 50 | 10 | 2.5 |
| 87 | MC-3 | DSPC | 5 | 40 | 16 | 2.5 |
| 88 | MC-3 | DSPC | 5 | 50 | 16 | 1.5 |
| 89 | MC-3 | DSPC | 5 | 60 | 10 | 2.5 |
| 90 | MC-3 | DSPC | 5 | 50 | 4 | 1.5 |
| 91 | MC-3 | DOPC | 5 | 60 | 16 | 1.5 |
| 92 | MC-3 | DOPC | 5 | 40 | 10 | 2.5 |
| 93 | MC-3 | DOPC | 5 | 50 | 10 | 0.5 |
| 94 | MC-3 | DOPC | 5 | 40 | 16 | 2.5 |
| 95 | MC-3 | DOPC | 5 | 60 | 10 | 0.5 |
| 96 | MC-3 | DOPC | 5 | 60 | 10 | 2.5 |
| 97 | MC-3 | DOPC | 5 | 60 | 10 | 1.5 |
| 98 | MC-3 | DOPC | 5 | 50 | 4 | 2.5 |
| 99 | MC-3 | DOPC | 5 | 40 | 4 | 1.5 |
| 100 | MC-3 | DOPC | 5 | 50 | 10 | 2.5 |
| 101 | MC-3 | DOPC | 5 | 50 | 16 | 0.5 |
| 102 | ALC-0315 | DSPC | 5 | 60 | 10 | 1.5 |
| 103 | ALC-0315 | DSPC | 5 | 50 | 4 | 2.5 |
| 104 | ALC-0315 | DSPC | 5 | 50 | 10 | 2.5 |
| 105 | ALC-0315 | DSPC | 5 | 50 | 16 | 0.5 |
| 106 | ALC-0315 | DSPC | 5 | 40 | 10 | 2.5 |
| 107 | ALC-0315 | DSPC | 5 | 40 | 10 | 0.5 |
| 108 | ALC-0315 | DSPC | 5 | 60 | 4 | 0.5 |
| 109 | ALC-0315 | DSPC | 5 | 60 | 4 | 2.5 |
| 110 | ALC-0315 | DSPC | 5 | 40 | 4 | 2.5 |
| 111 | ALC-0315 | DSPC | 5 | 50 | 16 | 1.5 |
| 112 | ALC-0315 | DSPC | 5 | 60 | 16 | 0.5 |
| 113 | ALC-0315 | DOPC | 5 | 60 | 16 | 1.5 |
| 114 | ALC-0315 | DOPC | 5 | 40 | 10 | 0.5 |
| 115 | ALC-0315 | DOPC | 5 | 40 | 4 | 0.5 |
| 116 | ALC-0315 | DOPC | 5 | 40 | 10 | 2.5 |
| 117 | ALC-0315 | DOPC | 5 | 50 | 4 | 0.5 |
| 118 | ALC-0315 | DOPC | 5 | 50 | 10 | 1.5 |
| 119 | ALC-0315 | DOPC | 5 | 50 | 4 | 1.5 |
| 120 | ALC-0315 | DOPC | 5 | 60 | 10 | 0.5 |
| 121 | ALC-0315 | DOPC | 5 | 40 | 4 | 1.5 |
| 122 | ALC-0315 | DOPC | 5 | 60 | 16 | 0.5 |
| 123 | CKK-E12 | DSPC | 5 | 50 | 16 | 2.5 |
| 124 | CKK-E12 | DSPC | 5 | 60 | 4 | 0.5 |
| 125 | CKK-E12 | DSPC | 5 | 50 | 10 | 1.5 |
| 126 | CKK-E12 | DSPC | 5 | 60 | 16 | 1.5 |
| 127 | CKK-E12 | DSPC | 5 | 50 | 16 | 1.5 |
| 128 | CKK-E12 | DSPC | 5 | 50 | 4 | 2.5 |
| 129 | CKK-E12 | DSPC | 5 | 50 | 10 | 0.5 |
| 130 | CKK-E12 | DSPC | 5 | 40 | 10 | 2.5 |
| 131 | CKK-E12 | DSPC | 5 | 40 | 10 | 0.5 |
| 132 | CKK-E12 | DSPC | 5 | 60 | 10 | 2.5 |
| 133 | CKK-E12 | DOPC | 5 | 40 | 4 | 2.5 |
| 134 | CKK-E12 | DOPC | 5 | 50 | 16 | 2.5 |
| 135 | CKK-E12 | DOPC | 5 | 40 | 4 | 1.5 |
| 136 | CKK-E12 | DOPC | 5 | 40 | 10 | 2.5 |
| 137 | CKK-E12 | DOPC | 5 | 50 | 10 | 2.5 |
| 138 | CKK-E12 | DOPC | 5 | 60 | 16 | 0.5 |
| 139 | CKK-E12 | DOPC | 5 | 60 | 4 | 1.5 |
| 140 | CKK-E12 | DOPC | 5 | 50 | 4 | 0.5 |
| 141 | CKK-E12 | DOPC | 5 | 50 | 16 | 0.5 |
| 142 | CKK-E12 | DOPC | 5 | 60 | 10 | 0.5 |
| 143 | SM-102 | DSPC | 10 | 50 | 10 | 1.5 |
| 144 | SM-102 | DSPC | 10 | 50 | 4 | 1.5 |
| 145 | SM-102 | DSPC | 10 | 60 | 16 | 1.5 |
| 146 | SM-102 | DSPC | 10 | 40 | 16 | 0.5 |
| 147 | SM-102 | DSPC | 10 | 40 | 10 | 2.5 |
| 148 | SM-102 | DSPC | 10 | 50 | 4 | 0.5 |
| 149 | SM-102 | DSPC | 10 | 40 | 16 | 2.5 |
| 150 | SM-102 | DSPC | 10 | 60 | 10 | 0.5 |
| 151 | SM-102 | DOPC | 10 | 40 | 16 | 1.5 |
| 152 | SM-102 | DOPC | 10 | 50 | 4 | 2.5 |
| 153 | SM-102 | DOPC | 10 | 60 | 10 | 1.5 |
| 154 | SM-102 | DOPC | 10 | 40 | 10 | 0.5 |
| 155 | SM-102 | DOPC | 10 | 50 | 16 | 2.5 |
| 156 | SM-102 | DOPC | 10 | 60 | 16 | 1.5 |
| 157 | SM-102 | DOPC | 10 | 50 | 10 | 2.5 |
| 158 | SM-102 | DOPC | 10 | 50 | 10 | 0.5 |
| 159 | MC-3 | DSPC | 10 | 50 | 4 | 2.5 |
| 160 | MC-3 | DSPC | 10 | 60 | 10 | 1.5 |
| 161 | MC-3 | DSPC | 10 | 50 | 4 | 0.5 |
| 162 | MC-3 | DSPC | 10 | 40 | 16 | 0.5 |
| 163 | MC-3 | DSPC | 10 | 40 | 10 | 0.5 |
| 164 | MC-3 | DSPC | 10 | 40 | 10 | 2.5 |
| 165 | MC-3 | DSPC | 10 | 60 | 4 | 2.5 |
| 166 | MC-3 | DOPC | 10 | 40 | 10 | 2.5 |
| 167 | MC-3 | DOPC | 10 | 60 | 16 | 0.5 |
| 168 | MC-3 | DOPC | 10 | 60 | 4 | 0.5 |
| 169 | MC-3 | DOPC | 10 | 60 | 4 | 1.5 |
| 170 | MC-3 | DOPC | 10 | 50 | 16 | 2.5 |
| 171 | MC-3 | DOPC | 10 | 50 | 4 | 0.5 |
| 172 | MC-3 | DOPC | 10 | 40 | 16 | 1.5 |
| 173 | MC-3 | DOPC | 10 | 40 | 4 | 1.5 |
| 174 | ALC-0315 | DSPC | 10 | 50 | 4 | 0.5 |
| 175 | ALC-0315 | DSPC | 10 | 50 | 16 | 2.5 |
| 176 | ALC-0315 | DSPC | 10 | 60 | 4 | 1.5 |
| 177 | ALC-0315 | DSPC | 10 | 60 | 10 | 1.5 |
| 178 | ALC-0315 | DSPC | 10 | 40 | 4 | 1.5 |
| 179 | ALC-0315 | DSPC | 10 | 50 | 10 | 2.5 |
| 180 | ALC-0315 | DSPC | 10 | 60 | 16 | 2.5 |
| 181 | ALC-0315 | DSPC | 10 | 40 | 10 | 0.5 |
| 182 | ALC-0315 | DOPC | 10 | 50 | 16 | 2.5 |
| 183 | ALC-0315 | DOPC | 10 | 40 | 16 | 1.5 |
| 184 | ALC-0315 | DOPC | 10 | 60 | 4 | 0.5 |
| 185 | ALC-0315 | DOPC | 10 | 50 | 4 | 0.5 |
| 186 | ALC-0315 | DOPC | 10 | 60 | 10 | 1.5 |
| 187 | ALC-0315 | DOPC | 10 | 60 | 16 | 2.5 |
| 188 | ALC-0315 | DOPC | 10 | 40 | 10 | 2.5 |
| 189 | ALC-0315 | DOPC | 10 | 60 | 4 | 2.5 |
| 190 | CKK-E12 | DSPC | 10 | 40 | 4 | 2.5 |
| 191 | CKK-E12 | DSPC | 10 | 40 | 4 | 0.5 |
| 192 | CKK-E12 | DSPC | 10 | 60 | 4 | 2.5 |
| 193 | CKK-E12 | DSPC | 10 | 40 | 4 | 1.5 |
| 194 | CKK-E12 | DSPC | 10 | 50 | 16 | 1.5 |
| 195 | CKK-E12 | DSPC | 10 | 60 | 16 | 2.5 |
| 196 | CKK-E12 | DSPC | 10 | 50 | 10 | 1.5 |
| 197 | CKK-E12 | DSPC | 10 | 40 | 10 | 1.5 |
| 198 | CKK-E12 | DOPC | 10 | 50 | 4 | 2.5 |
| 199 | CKK-E12 | DOPC | 10 | 60 | 4 | 0.5 |
| 200 | CKK-E12 | DOPC | 10 | 40 | 16 | 1.5 |
| 201 | CKK-E12 | DOPC | 10 | 60 | 16 | 1.5 |
| 202 | CKK-E12 | DOPC | 10 | 60 | 10 | 2.5 |
| 203 | CKK-E12 | DOPC | 10 | 60 | 16 | 0.5 |

Table S2. Top 25 formulations selected for in vitro validation based on scoring results

| No | Ionizable Lipid Type | Helper Lipid Type | Ionizable Lipid Concentration (mM) | Ionizable Lipid Ratio (%) | Helper Lipid Ratio (%) | PEG Lipid Ratio (%) |
| --- | --- | --- | --- | --- | --- | --- |
| 1 | ALC-0315 | DSPC | 2.5 | 60 | 4 | 1.5 |
| 2 | ALC-0315 | DSPC | 5 | 60 | 4 | 1.5 |
| 3 | ALC-0315 | DOPC | 5 | 50 | 4 | 1.5 |
| 4 | ALC-0315 | DSPC | 5 | 60 | 10 | 0.5 |
| 5 | ALC-0315 | DOPC | 5 | 40 | 10 | 1.5 |
| 6 | ALC-0315 | DOPC | 5 | 40 | 4 | 1.5 |
| 7 | ALC-0315 | DSPC | 5 | 60 | 4 | 0.5 |
| 8 | SM-102 | DOPC | 5 | 60 | 10 | 0.5 |
| 9 | SM-102 | DSPC | 2.5 | 40 | 10 | 0.5 |
| 10 | SM-102 | DSPC | 2.5 | 50 | 10 | 0.5 |
| 11 | SM-102 | DSPC | 2.5 | 50 | 16 | 0.5 |
| 12 | SM-102 | DSPC | 1.25 | 50 | 10 | 0.5 |
| 13 | SM-102 | DOPC | 5 | 50 | 10 | 0.5 |
| 14 | MC-3 | DSPC | 2.5 | 60 | 4 | 1.5 |
| 15 | MC-3 | DSPC | 5 | 60 | 4 | 1.5 |
| 16 | MC-3 | DOPC | 5 | 50 | 4 | 1.5 |
| 17 | MC-3 | DSPC | 5 | 50 | 4 | 1.5 |
| 18 | MC-3 | DOPC | 5 | 40 | 4 | 1.5 |
| 19 | MC-3 | DSPC | 2.5 | 60 | 4 | 2.5 |
| 20 | CKK-E12 | DSPC | 1.25 | 60 | 4 | 2.5 |
| 21 | CKK-E12 | DSPC | 10 | 60 | 4 | 2.5 |
| 22 | CKK-E12 | DOPC | 10 | 60 | 4 | 2.5 |
| 23 | CKK-E12 | DOPC | 10 | 60 | 10 | 2.5 |
| 24 | CKK-E12 | DOPC | 10 | 50 | 4 | 1.5 |
| 25 | CKK-E12 | DOPC | 10 | 40 | 4 | 1.5 |

Table S3. Formulations of the 4 selected LNP for in vivo biodistribution evolution

| No | Ionizable Lipid Type | Helper Lipid Type | Ionizable Lipid Concentration (mM) | Ionizable Lipid Ratio (%) | Helper Lipid Ratio (%) | PEG Lipid Ratio (%) |
| --- | --- | --- | --- | --- | --- | --- |
| 1 | ALC-0315 | DOPC | 5 | 40 | 10 | 1.5 |
| 2 | SM-102 | DOPC | 5 | 50 | 10 | 0.5 |
| 3 | MC-3 | DSPC | 2.5 | 60 | 4 | 2.5 |
| 4 | CKK-E12 | DOPC | 10 | 50 | 4 | 1.5 |
